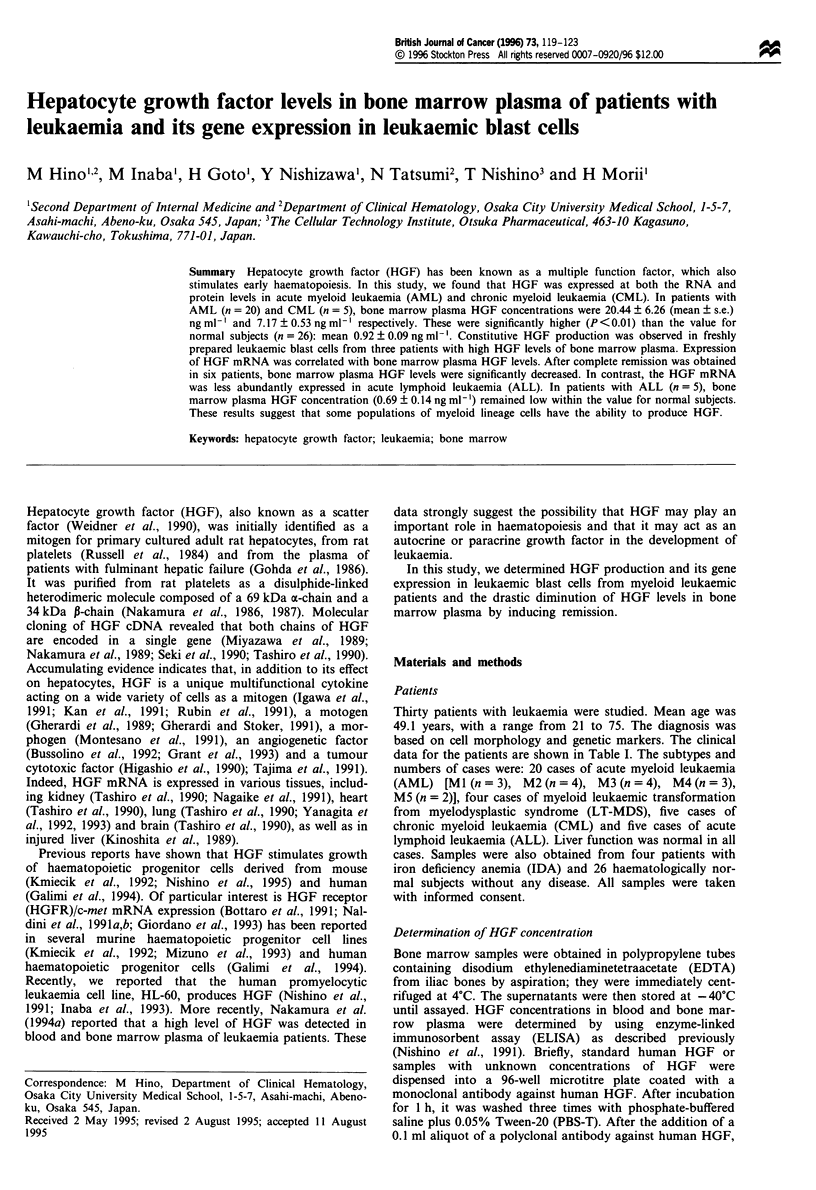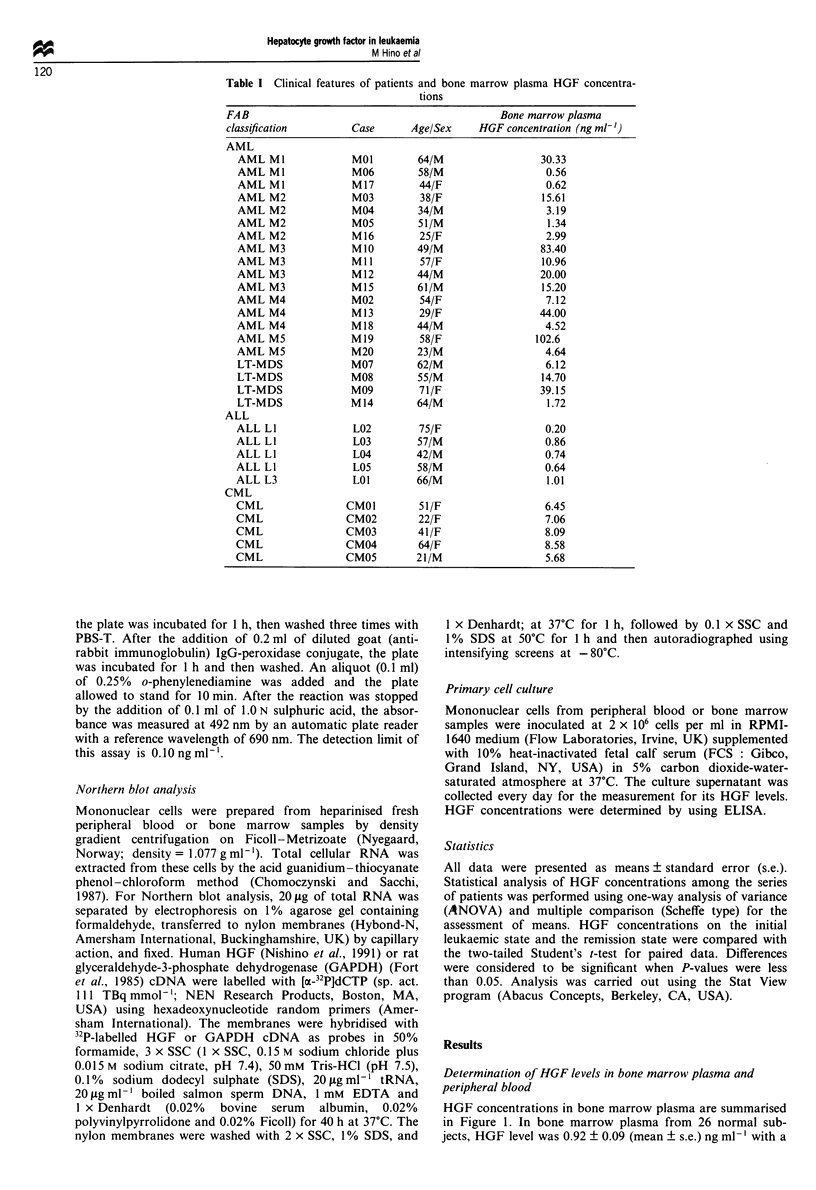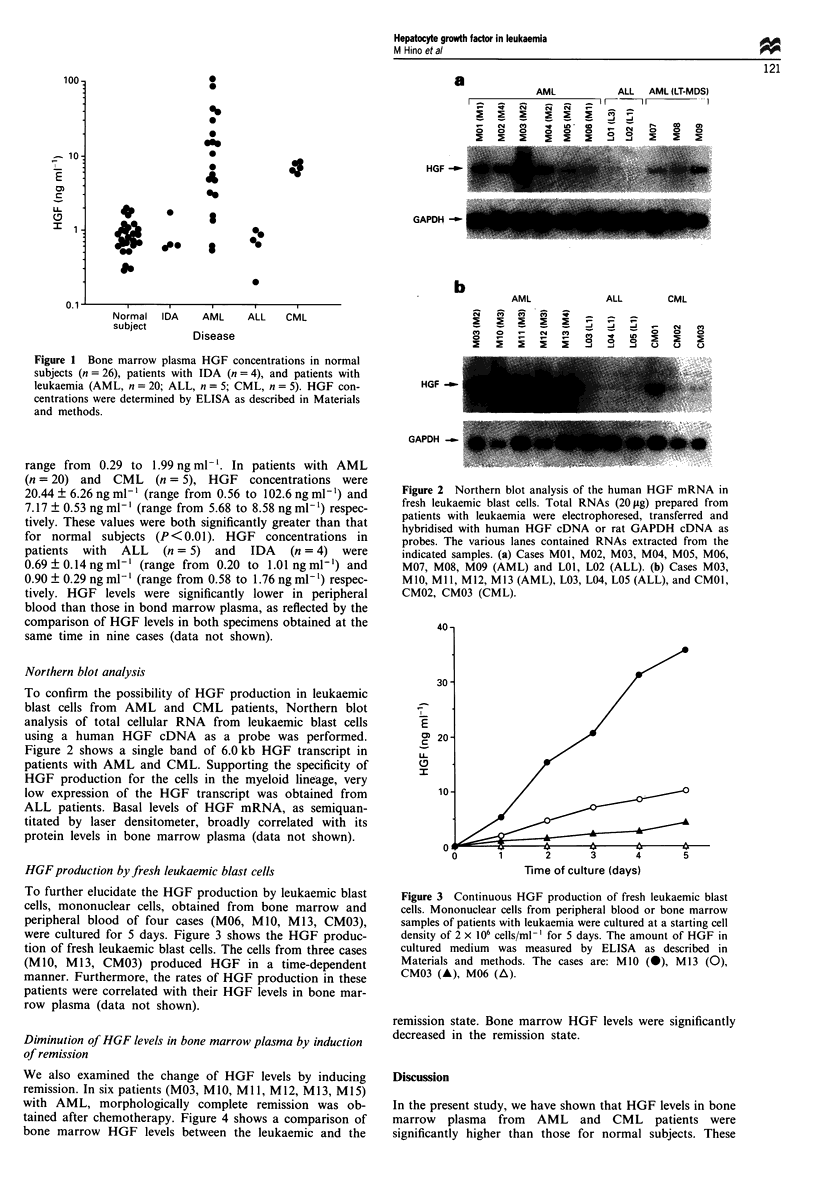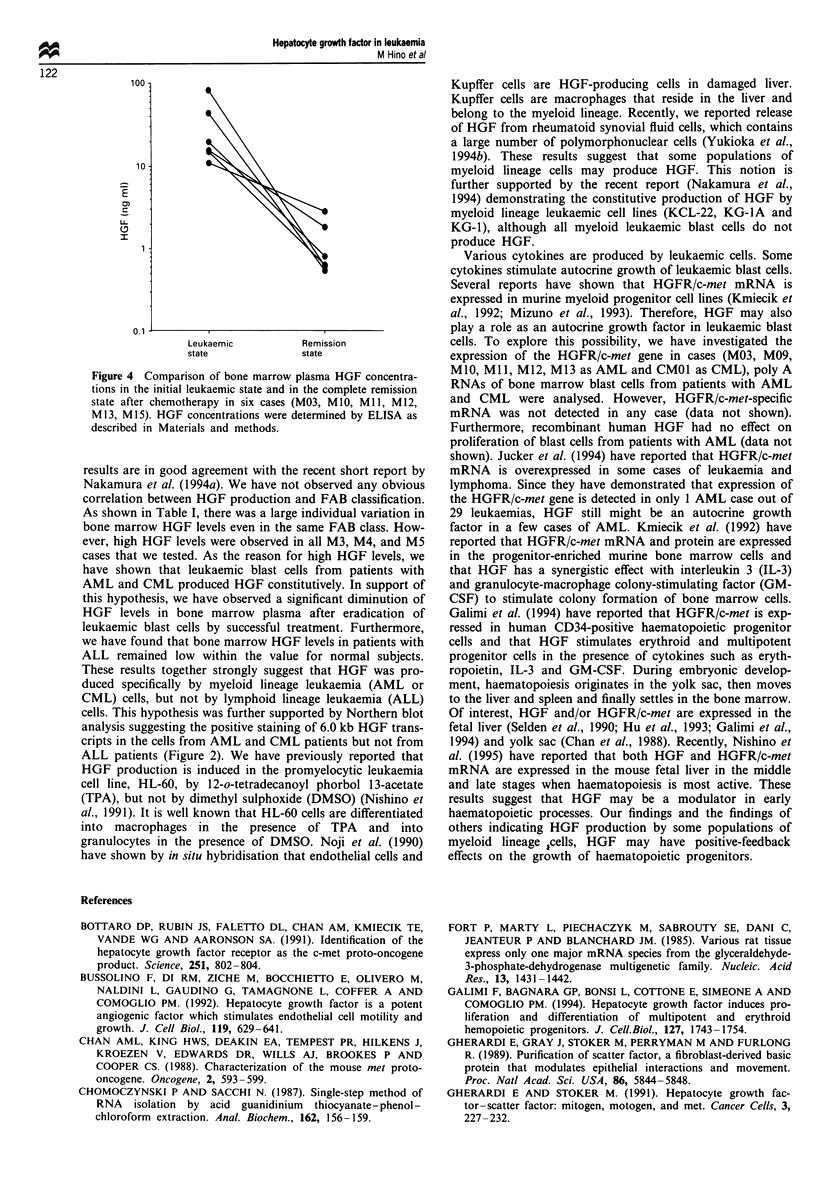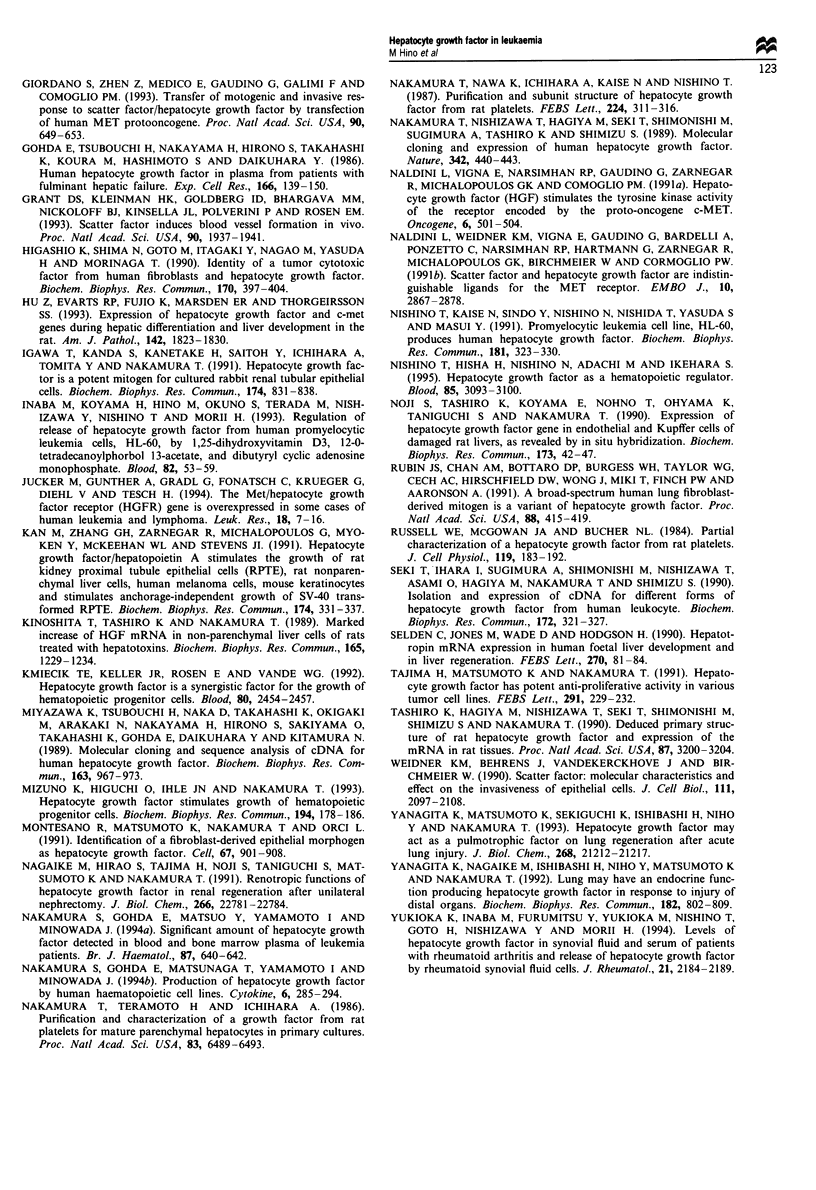# Hepatocyte growth factor levels in bone marrow plasma of patients with leukaemia and its gene expression in leukaemic blast cells.

**DOI:** 10.1038/bjc.1996.22

**Published:** 1996-01

**Authors:** M. Hino, M. Inaba, H. Goto, Y. Nishizawa, N. Tatsumi, T. Nishino, H. Morii

**Affiliations:** Second Department of Internal Medicine, Osaka City University Medical School, Japan.

## Abstract

**Images:**


British Journal of Cancer (1996) 73, 119–123
© 1996 Stockton Press All rights reserved 0007–0920/96 $12.00

### Hepatocyte growth factor levels in bone marrow plasma of patients with leukaemia and its gene expression in leukaemic blast cells

M Hino[1,2], M Inaba[1], H Goto[1], Y Nishizawa[1], N Tatsumi[2], T Nishino[3] and H Morii[1]

[1]*Second Department of Internal Medicine and* [2]*Department of Clinical Hematology, Osaka City University Medical School, 1-5-7, Asahi-machi, Abeno-ku, Osaka 545, Japan;* [3]*The Cellular Technology Institute, Otsuka Pharmaceutical, 463-10 Kagasuno, Kawauchi-cho, Tokushima, 771-01, Japan.*

**Summary** Hepatocyte growth factor (HGF) has been known as a multiple function factor, which also stimulates early haematopoiesis. In this study, we found that HGF was expressed at both the RNA and protein levels in acute myeloid leukaemia (AML) and chronic myeloid leukaemia (CML). In patients with AML ($n = 20$) and CML ($n = 5$), bone marrow plasma HGF concentrations were $20.44 \pm 6.26$ (mean ± s.e.) ng $ml^{-1}$ and $7.17 \pm 0.53$ ng $ml^{-1}$ respectively. These were significantly higher ($P < 0.01$) than the value for normal subjects ($n = 26$): mean $0.92 \pm 0.09$ ng $ml^{-1}$. Constitutive HGF production was observed in freshly prepared leukaemic blast cells from three patients with high HGF levels of bone marrow plasma. Expression of HGF mRNA was correlated with bone marrow plasma HGF levels. After complete remission was obtained in six patients, bone marrow plasma HGF levels were significantly decreased. In contrast, the HGF mRNA was less abundantly expressed in acute lymphoid leukaemia (ALL). In patients with ALL ($n = 5$), bone marrow plasma HGF concentration ($0.69 \pm 0.14$ ng $ml^{-1}$) remained low within the value for normal subjects. These results suggest that some populations of myeloid lineage cells have the ability to produce HGF.

**Keywords:** hepatocyte growth factor; leukaemia; bone marrow

Hepatocyte growth factor (HGF), also known as a scatter factor (Weidner *et al.*, 1990), was initially identified as a mitogen for primary cultured adult rat hepatocytes, from rat platelets (Russell *et al.*, 1984) and from the plasma of patients with fulminant hepatic failure (Gohda *et al.*, 1986). It was purified from rat platelets as a disulphide-linked heterodimeric molecule composed of a 69 kDa α-chain and a 34 kDa β-chain (Nakamura *et al.*, 1986, 1987). Molecular cloning of HGF cDNA revealed that both chains of HGF are encoded in a single gene (Miyazawa *et al.*, 1989; Nakamura *et al.*, 1989; Seki *et al.*, 1990; Tashiro *et al.*, 1990). Accumulating evidence indicates that, in addition to its effect on hepatocytes, HGF is a unique multifunctional cytokine acting on a wide variety of cells as a mitogen (Igawa *et al.*, 1991; Kan *et al.*, 1991; Rubin *et al.*, 1991), a motogen (Gherardi *et al.*, 1989; Gherardi and Stoker, 1991), a morphogen (Montesano *et al.*, 1991), an angiogenetic factor (Bussolino *et al.*, 1992; Grant *et al.*, 1993) and a tumour cytotoxic factor (Higashio *et al.*, 1990); Tajima *et al.*, 1991). Indeed, HGF mRNA is expressed in various tissues, including kidney (Tashiro *et al.*, 1990; Nagaike *et al.*, 1991), heart (Tashiro *et al.*, 1990), lung (Tashiro *et al.*, 1990; Yanagita *et al.*, 1992, 1993) and brain (Tashiro *et al.*, 1990), as well as in injured liver (Kinoshita *et al.*, 1989).

Previous reports have shown that HGF stimulates growth of haematopoietic progenitor cells derived from mouse (Kmiecik *et al.*, 1992; Nishino *et al.*, 1995) and human (Galimi *et al.*, 1994). Of particular interest is HGF receptor (HGFR)/c-*met* mRNA expression (Bottaro *et al.*, 1991; Naldini *et al.*, 1991*a*,*b*; Giordano *et al.*, 1993) has been reported in several murine haematopoietic progenitor cell lines (Kmiecik *et al.*, 1992; Mizuno *et al.*, 1993) and human haematopoietic progenitor cells (Galimi *et al.*, 1994). Recently, we reported that the human promyelocytic leukaemia cell line, HL-60, produces HGF (Nishino *et al.*, 1991; Inaba *et al.*, 1993). More recently, Nakamura *et al.* (1994*a*) reported that a high level of HGF was detected in blood and bone marrow plasma of leukaemia patients. These data strongly suggest the possibility that HGF may play an important role in haematopoiesis and that it may act as an autocrine or paracrine growth factor in the development of leukaemia.

In this study, we determined HGF production and its gene expression in leukaemic blast cells from myeloid leukaemic patients and the drastic diminution of HGF levels in bone marrow plasma by inducing remission.

### Materials and methods

### *Patients*

Thirty patients with leukaemia were studied. Mean age was 49.1 years, with a range from 21 to 75. The diagnosis was based on cell morphology and genetic markers. The clinical data for the patients are shown in Table I. The subtypes and numbers of cases were: 20 cases of acute myeloid leukaemia (AML) [M1 ($n = 3$), M2 ($n = 4$), M3 ($n = 4$), M4 ($n = 3$), M5 ($n = 2$)], four cases of myeloid leukaemic transformation from myelodysplastic syndrome (LT-MDS), five cases of chronic myeloid leukaemia (CML) and five cases of acute lymphoid leukaemia (ALL). Liver function was normal in all cases. Samples were also obtained from four patients with iron deficiency anemia (IDA) and 26 haematologically normal subjects without any disease. All samples were taken with informed consent.

### *Determination of HGF concentration*

Bone marrow samples were obtained in polypropylene tubes containing disodium ethylenediaminetetraacetate (EDTA) from iliac bones by aspiration; they were immediately centrifuged at 4°C. The supernatants were then stored at −40°C until assayed. HGF concentrations in blood and bone marrow plasma were determined by using enzyme-linked immunosorbent assay (ELISA) as described previously (Nishino *et al.*, 1991). Briefly, standard human HGF or samples with unknown concentrations of HGF were dispensed into a 96-well microtitre plate coated with a monoclonal antibody against human HGF. After incubation for 1 h, it was washed three times with phosphate-buffered saline plus 0.05% Tween-20 (PBS-T). After the addition of a 0.1 ml aliquot of a polyclonal antibody against human HGF,

Correspondence: M Hino, Department of Clinical Hematology, Osaka City University Medical School, 1-5-7, Asahi-machi, Abeno-ku, Osaka 545, Japan.
Received 2 May 1995; revised 2 August 1995; accepted 11 August 1995

**Table I** Clinical features of patients and bone marrow plasma HGF concentrations

| *FAB classification* | *Case* | *Age/Sex* | *Bone marrow plasma HGF concentration (ng ml$^{-1}$)* |
|---|---|---|---|
| AML | | | |
| AML M1 | M01 | 64/M | 30.33 |
| AML M1 | M06 | 58/M | 0.56 |
| AML M1 | M17 | 44/F | 0.62 |
| AML M2 | M03 | 38/F | 15.61 |
| AML M2 | M04 | 34/M | 3.19 |
| AML M2 | M05 | 51/M | 1.34 |
| AML M2 | M16 | 25/F | 2.99 |
| AML M3 | M10 | 49/M | 83.40 |
| AML M3 | M11 | 57/F | 10.96 |
| AML M3 | M12 | 44/M | 20.00 |
| AML M3 | M15 | 61/M | 15.20 |
| AML M4 | M02 | 54/F | 7.12 |
| AML M4 | M13 | 29/F | 44.00 |
| AML M4 | M18 | 44/M | 4.52 |
| AML M5 | M19 | 58/F | 102.6 |
| AML M5 | M20 | 23/M | 4.64 |
| LT-MDS | M07 | 62/M | 6.12 |
| LT-MDS | M08 | 55/M | 14.70 |
| LT-MDS | M09 | 71/F | 39.15 |
| LT-MDS | M14 | 64/M | 1.72 |
| ALL | | | |
| ALL L1 | L02 | 75/F | 0.20 |
| ALL L1 | L03 | 57/M | 0.86 |
| ALL L1 | L04 | 42/M | 0.74 |
| ALL L1 | L05 | 58/M | 0.64 |
| ALL L3 | L01 | 66/M | 1.01 |
| CML | | | |
| CML | CM01 | 51/F | 6.45 |
| CML | CM02 | 22/F | 7.06 |
| CML | CM03 | 41/F | 8.09 |
| CML | CM04 | 64/F | 8.58 |
| CML | CM05 | 21/M | 5.68 |

the plate was incubated for 1 h, then washed three times with PBS-T. After the addition of 0.2 ml of diluted goat (anti-rabbit immunoglobulin) IgG-peroxidase conjugate, the plate was incubated for 1 h and then washed. An aliquot (0.1 ml) of 0.25% *o*-phenylenediamine was added and the plate allowed to stand for 10 min. After the reaction was stopped by the addition of 0.1 ml of 1.0 N sulphuric acid, the absorbance was measured at 492 nm by an automatic plate reader with a reference wavelength of 690 nm. The detection limit of this assay is 0.10 ng ml$^{-1}$.

*Northern blot analysis*

Mononuclear cells were prepared from heparinised fresh peripheral blood or bone marrow samples by density gradient centrifugation on Ficoll–Metrizoate (Nyegaard, Norway; density = 1.077 g ml$^{-1}$). Total cellular RNA was extracted from these cells by the acid guanidium–thiocyanate phenol–chloroform method (Chomoczynski and Sacchi, 1987). For Northern blot analysis, 20 μg of total RNA was separated by electrophoresis on 1% agarose gel containing formaldehyde, transferred to nylon membranes (Hybond-N, Amersham International, Buckinghamshire, UK) by capillary action, and fixed. Human HGF (Nishino *et al.*, 1991) or rat glyceraldehyde-3-phosphate dehydrogenase (GAPDH) (Fort *et al.*, 1985) cDNA were labelled with [α-$^{32}$P]dCTP (sp. act. 111 TBq mmol$^{-1}$; NEN Research Products, Boston, MA, USA) using hexadeoxynucleotide random primers (Amersham International). The membranes were hybridised with $^{32}$P-labelled HGF or GAPDH cDNA as probes in 50% formamide, 3 × SSC (1 × SSC, 0.15 M sodium chloride plus 0.015 M sodium citrate, pH 7.4), 50 mM Tris-HCl (pH 7.5), 0.1% sodium dodecyl sulphate (SDS), 20 μg ml$^{-1}$ tRNA, 20 μg ml$^{-1}$ boiled salmon sperm DNA, 1 mM EDTA and 1 × Denhardt (0.02% bovine serum albumin, 0.02% polyvinylpyrrolidone and 0.02% Ficoll) for 40 h at 37°C. The nylon membranes were washed with 2 × SSC, 1% SDS, and 1 × Denhardt; at 37°C for 1 h, followed by 0.1 × SSC and 1% SDS at 50°C for 1 h and then autoradiographed using intensifying screens at −80°C.

*Primary cell culture*

Mononuclear cells from peripheral blood or bone marrow samples were inoculated at 2 × 10$^{6}$ cells per ml in RPMI-1640 medium (Flow Laboratories, Irvine, UK) supplemented with 10% heat-inactivated fetal calf serum (FCS : Gibco, Grand Island, NY, USA) in 5% carbon dioxide-water-saturated atmosphere at 37°C. The culture supernatant was collected every day for the measurement for its HGF levels. HGF concentrations were determined by using ELISA.

*Statistics*

All data were presented as means ± standard error (s.e.). Statistical analysis of HGF concentrations among the series of patients was performed using one-way analysis of variance (ANOVA) and multiple comparison (Scheffe type) for the assessment of means. HGF concentrations on the initial leukaemic state and the remission state were compared with the two-tailed Student's *t*-test for paired data. Differences were considered to be significant when *P*-values were less than 0.05. Analysis was carried out using the Stat View program (Abacus Concepts, Berkeley, CA, USA).

### Results

*Determination of HGF levels in bone marrow plasma and peripheral blood*

HGF concentrations in bone marrow plasma are summarised in Figure 1. In bone marrow plasma from 26 normal subjects, HGF level was 0.92 ± 0.09 (mean ± s.e.) ng ml$^{-1}$ with a

**Figure 1** Bone marrow plasma HGF concentrations in normal subjects ($n = 26$), patients with IDA ($n = 4$), and patients with leukaemia (AML, $n = 20$; ALL, $n = 5$; CML, $n = 5$). HGF concentrations were determined by ELISA as described in Materials and methods.

range from 0.29 to 1.99 ng $ml^{-1}$. In patients with AML ($n = 20$) and CML ($n = 5$), HGF concentrations were $20.44 \pm 6.26$ ng $ml^{-1}$ (range from 0.56 to 102.6 ng $ml^{-1}$) and $7.17 \pm 0.53$ ng $ml^{-1}$ (range from 5.68 to 8.58 ng $ml^{-1}$) respectively. These values were both significantly greater than that for normal subjects ($P < 0.01$). HGF concentrations in patients with ALL ($n = 5$) and IDA ($n = 4$) were $0.69 \pm 0.14$ ng $ml^{-1}$ (range from 0.20 to 1.01 ng $ml^{-1}$) and $0.90 \pm 0.29$ ng $ml^{-1}$ (range from 0.58 to 1.76 ng $ml^{-1}$) respectively. HGF levels were significantly lower in peripheral blood than those in bond marrow plasma, as reflected by the comparison of HGF levels in both specimens obtained at the same time in nine cases (data not shown).

*Northern blot analysis*

To confirm the possibility of HGF production in leukaemic blast cells from AML and CML patients, Northern blot analysis of total cellular RNA from leukaemic blast cells using a human HGF cDNA as a probe was performed. Figure 2 shows a single band of 6.0 kb HGF transcript in patients with AML and CML. Supporting the specificity of HGF production for the cells in the myeloid lineage, very low expression of the HGF transcript was obtained from ALL patients. Basal levels of HGF mRNA, as semiquantitated by laser densitometer, broadly correlated with its protein levels in bone marrow plasma (data not shown).

*HGF production by fresh leukaemic blast cells*

To further elucidate the HGF production by leukaemic blast cells, mononuclear cells, obtained from bone marrow and peripheral blood of four cases (M06, M10, M13, CM03), were cultured for 5 days. Figure 3 shows the HGF production of fresh leukaemic blast cells. The cells from three cases (M10, M13, CM03) produced HGF in a time-dependent manner. Furthermore, the rates of HGF production in these patients were correlated with their HGF levels in bone marrow plasma (data not shown).

*Diminution of HGF levels in bone marrow plasma by induction of remission*

We also examined the change of HGF levels by inducing remission. In six patients (M03, M10, M11, M12, M13, M15) with AML, morphologically complete remission was obtained after chemotherapy. Figure 4 shows a comparison of bone marrow HGF levels between the leukaemic and the

**Figure 2** Northern blot analysis of the human HGF mRNA in fresh leukaemic blast cells. Total RNAs (20 μg) prepared from patients with leukaemia were electrophoresed, transferred and hybridised with human HGF cDNA or rat GAPDH cDNA as probes. The various lanes contained RNAs extracted from the indicated samples. (**a**) Cases M01, M02, M03, M04, M05, M06, M07, M08, M09 (AML) and L01, L02 (ALL). (**b**) Cases M03, M10, M11, M12, M13 (AML), L03, L04, L05 (ALL), and CM01, CM02, CM03 (CML).

**Figure 3** Continuous HGF production of fresh leukaemic blast cells. Mononuclear cells from peripheral blood or bone marrow samples of patients with leukaemia were cultured at a starting cell density of $2 \times 10^6$ cells/$ml^{-1}$ for 5 days. The amount of HGF in cultured medium was measured by ELISA as described in Materials and methods. The cases are: M10 (●), M13 (○), CM03 (▲), M06 (△).

remission state. Bone marrow HGF levels were significantly decreased in the remission state.

### Discussion

In the present study, we have shown that HGF levels in bone marrow plasma from AML and CML patients were significantly higher than those for normal subjects. These

**Figure 4** Comparison of bone marrow plasma HGF concentrations in the initial leukaemic state and in the complete remission state after chemotherapy in six cases (M03, M10, M11, M12, M13, M15). HGF concentrations were determined by ELISA as described in Materials and methods.

results are in good agreement with the recent short report by Nakamura *et al.* (1994*a*). We have not observed any obvious correlation between HGF production and FAB classification. As shown in Table I, there was a large individual variation in bone marrow HGF levels even in the same FAB class. However, high HGF levels were observed in all M3, M4, and M5 cases that we tested. As the reason for high HGF levels, we have shown that leukaemic blast cells from patients with AML and CML produced HGF constitutively. In support of this hypothesis, we have observed a significant diminution of HGF levels in bone marrow plasma after eradication of leukaemic blast cells by successful treatment. Furthermore, we have found that bone marrow HGF levels in patients with ALL remained low within the value for normal subjects. These results together strongly suggest that HGF was produced specifically by myeloid lineage leukaemia (AML or CML) cells, but not by lymphoid lineage leukaemia (ALL) cells. This hypothesis was further supported by Northern blot analysis suggesting the positive staining of 6.0 kb HGF transcripts in the cells from AML and CML patients but not from ALL patients (Figure 2). We have previously reported that HGF production is induced in the promyelocytic leukaemia cell line, HL-60, by 12-*o*-tetradecanoyl phorbol 13-acetate (TPA), but not by dimethyl sulphoxide (DMSO) (Nishino *et al.*, 1991). It is well known that HL-60 cells are differentiated into macrophages in the presence of TPA and into granulocytes in the presence of DMSO. Noji *et al.* (1990) have shown by *in situ* hybridisation that endothelial cells and Kupffer cells are HGF-producing cells in damaged liver. Kupffer cells are macrophages that reside in the liver and belong to the myeloid lineage. Recently, we reported release of HGF from rheumatoid synovial fluid cells, which contains a large number of polymorphonuclear cells (Yukioka *et al.*, 1994*b*). These results suggest that some populations of myeloid lineage cells may produce HGF. This notion is further supported by the recent report (Nakamura *et al.*, 1994) demonstrating the constitutive production of HGF by myeloid lineage leukaemic cell lines (KCL-22, KG-1A and KG-1), although all myeloid leukaemic blast cells do not produce HGF.

Various cytokines are produced by leukaemic cells. Some cytokines stimulate autocrine growth of leukaemic blast cells. Several reports have shown that HGFR/c-*met* mRNA is expressed in murine myeloid progenitor cell lines (Kmiecik *et al.*, 1992; Mizuno *et al.*, 1993). Therefore, HGF may also play a role as an autocrine growth factor in leukaemic blast cells. To explore this possibility, we have investigated the expression of the HGFR/c-*met* gene in cases (M03, M09, M10, M11, M12, M13 as AML and CM01 as CML), poly A RNAs of bone marrow blast cells from patients with AML and CML were analysed. However, HGFR/c-*met*-specific mRNA was not detected in any case (data not shown). Furthermore, recombinant human HGF had no effect on proliferation of blast cells from patients with AML (data not shown). Jucker *et al.* (1994) have reported that HGFR/c-*met* mRNA is overexpressed in some cases of leukaemia and lymphoma. Since they have demonstrated that expression of the HGFR/c-*met* gene is detected in only 1 AML case out of 29 leukaemias, HGF still might be an autocrine growth factor in a few cases of AML. Kmiecik *et al.* (1992) have reported that HGFR/c-*met* mRNA and protein are expressed in the progenitor-enriched murine bone marrow cells and that HGF has a synergistic effect with interleukin 3 (IL-3) and granulocyte-macrophage colony-stimulating factor (GM-CSF) to stimulate colony formation of bone marrow cells. Galimi *et al.* (1994) have reported that HGFR/c-*met* is expressed in human CD34-positive haematopoietic progenitor cells and that HGF stimulates erythroid and multipotent progenitor cells in the presence of cytokines such as erythropoietin, IL-3 and GM-CSF. During embryonic development, haematopoiesis originates in the yolk sac, then moves to the liver and spleen and finally settles in the bone marrow. Of interest, HGF and/or HGFR/c-*met* are expressed in the fetal liver (Selden *et al.*, 1990; Hu *et al.*, 1993; Galimi *et al.*, 1994) and yolk sac (Chan *et al.*, 1988). Recently, Nishino *et al.* (1995) have reported that both HGF and HGFR/c-*met* mRNA are expressed in the mouse fetal liver in the middle and late stages when haematopoiesis is most active. These results suggest that HGF may be a modulator in early haematopoietic processes. Our findings and the findings of others indicating HGF production by some populations of myeloid lineage cells, HGF may have positive-feedback effects on the growth of haematopoietic progenitors.

### References

BOTTARO DP, RUBIN JS, FALETTO DL, CHAN AM, KMIECIK TE, VANDE WG AND AARONSON SA. (1991). Identification of the hepatocyte growth factor receptor as the c-met proto-oncogene product. *Science*, **251**, 802-804.

BUSSOLINO F, DI RM, ZICHE M, BOCCHIETTO E, OLIVERO M, NALDINI L, GAUDINO G, TAMAGNONE L, COFFER A AND COMOGLIO PM. (1992). Hepatocyte growth factor is a potent angiogenic factor which stimulates endothelial cell motility and growth. *J. Cell Biol.*, **119**, 629-641.

CHAN AML, KING HWS, DEAKIN EA, TEMPEST PR, HILKENS J, KROEZEN V, EDWARDS DR, WILLS AJ, BROOKES P AND COOPER CS. (1988). Characterization of the mouse *met* proto-oncogene. *Oncogene*, **2**, 593-599.

CHOMOCZYNSKI P AND SACCHI N. (1987). Single-step method of RNA isolation by acid guanidinium thiocyanate-phenol-chloroform extraction. *Anal. Biochem.*, **162**, 156-159.

FORT P, MARTY L, PIECHACZYK M, SABROUTY SE, DANI C, JEANTEUR P AND BLANCHARD JM. (1985). Various rat tissue express only one major mRNA species from the glyceraldehyde-3-phosphate-dehydrogenase multigenetic family. *Nucleic. Acid Res.*, **13**, 1431-1442.

GALIMI F, BAGNARA GP, BONSI L, COTTONE E, SIMEONE A AND COMOGLIO PM. (1994). Hepatocyte growth factor induces proliferation and differentiation of multipotent and erythroid hemopoietic progenitors. *J. Cell.Biol.*, **127**, 1743-1754.

GHERARDI E, GRAY J, STOKER M, PERRYMAN M AND FURLONG R. (1989). Purification of scatter factor, a fibroblast-derived basic protein that modulates epithelial interactions and movement. *Proc. Natl Acad. Sci. USA*, **86**, 5844-5848.

GHERARDI E AND STOKER M. (1991). Hepatocyte growth factor-scatter factor: mitogen, motogen, and met. *Cancer Cells*, **3**, 227-232.

GIORDANO S, ZHEN Z, MEDICO E, GAUDINO G, GALIMI F AND COMOGLIO PM. (1993). Transfer of motogenic and invasive response to scatter factor/hepatocyte growth factor by transfection of human MET protooncogene. *Proc. Natl Acad. Sci. USA*, **90**, 649-653.

GOHDA E, TSUBOUCHI H, NAKAYAMA H, HIRONO S, TAKAHASHI K, KOURA M, HASHIMOTO S AND DAIKUHARA Y. (1986). Human hepatocyte growth factor in plasma from patients with fulminant hepatic failure. *Exp. Cell Res.*, **166**, 139-150.

GRANT DS, KLEINMAN HK, GOLDBERG ID, BHARGAVA MM, NICKOLOFF BJ, KINSELLA JL, POLVERINI P AND ROSEN EM. (1993). Scatter factor induces blood vessel formation in vivo. *Proc. Natl Acad. Sci. USA*, **90**, 1937-1941.

HIGASHIO K, SHIMA N, GOTO M, ITAGAKI Y, NAGAO M, YASUDA H AND MORINAGA T. (1990). Identity of a tumor cytotoxic factor from human fibroblasts and hepatocyte growth factor. *Biochem. Biophys. Res. Commun.*, **170**, 397-404.

HU Z, EVARTS RP, FUJIO K, MARSDEN ER AND THORGEIRSSON SS. (1993). Expression of hepatocyte growth factor and c-met genes during hepatic differentiation and liver development in the rat. *Am. J. Pathol.*, **142**, 1823-1830.

IGAWA T, KANDA S, KANETAKE H, SAITOH Y, ICHIHARA A, TOMITA Y AND NAKAMURA T. (1991). Hepatocyte growth factor is a potent mitogen for cultured rabbit renal tubular epithelial cells. *Biochem. Biophys. Res. Commun.*, **174**, 831-838.

INABA M, KOYAMA H, HINO M, OKUNO S, TERADA M, NISHIZAWA Y, NISHINO T AND MORII H. (1993). Regulation of release of hepatocyte growth factor from human promyelocytic leukemia cells, HL-60, by 1,25-dihydroxyvitamin D3, 12-0-tetradecanoylphorbol 13-acetate, and dibutyryl cyclic adenosine monophosphate. *Blood*, **82**, 53-59.

JUCKER M, GUNTHER A, GRADL G, FONATSCH C, KRUEGER G, DIEHL V AND TESCH H. (1994). The Met/hepatocyte growth factor receptor (HGFR) gene is overexpressed in some cases of human leukemia and lymphoma. *Leuk. Res.*, **18**, 7-16.

KAN M, ZHANG GH, ZARNEGAR R, MICHALOPOULOS G, MYOKEN Y, MCKEEHAN WL AND STEVENS JI. (1991). Hepatocyte growth factor/hepatopoietin A stimulates the growth of rat kidney proximal tubule epithelial cells (RPTE), rat nonparenchymal liver cells, human melanoma cells, mouse keratinocytes and stimulates anchorage-independent growth of SV-40 transformed RPTE. *Biochem. Biophys. Res. Commun.*, **174**, 331-337.

KINOSHITA T, TASHIRO K AND NAKAMURA T. (1989). Marked increase of HGF mRNA in non-parenchymal liver cells of rats treated with hepatotoxins. *Biochem. Biophys. Res. Commun.*, **165**, 1229-1234.

KMIECIK TE, KELLER JR, ROSEN E AND VANDE WG. (1992). Hepatocyte growth factor is a synergistic factor for the growth of hematopoietic progenitor cells. *Blood*, **80**, 2454-2457.

MIYAZAWA K, TSUBOUCHI H, NAKA D, TAKAHASHI K, OKIGAKI M, ARAKAKI N, NAKAYAMA H, HIRONO S, SAKIYAMA O, TAKAHASHI K, GOHDA E, DAIKUHARA Y AND KITAMURA N. (1989). Molecular cloning and sequence analysis of cDNA for human hepatocyte growth factor. *Biochem. Biophys. Res. Commun.*, **163**, 967-973.

MIZUNO K, HIGUCHI O, IHLE JN AND NAKAMURA T. (1993). Hepatocyte growth factor stimulates growth of hematopoietic progenitor cells. *Biochem. Biophys. Res. Commun.*, **194**, 178-186.

MONTESANO R, MATSUMOTO K, NAKAMURA T AND ORCI L. (1991). Identification of a fibroblast-derived epithelial morphogen as hepatocyte growth factor. *Cell*, **67**, 901-908.

NAGAIKE M, HIRAO S, TAJIMA H, NOJI S, TANIGUCHI S, MATSUMOTO K AND NAKAMURA T. (1991). Renotropic functions of hepatocyte growth factor in renal regeneration after unilateral nephrectomy. *J. Biol. Chem.*, **266**, 22781-22784.

NAKAMURA S, GOHDA E, MATSUO Y, YAMAMOTO I AND MINOWADA J. (1994*a*). Significant amount of hepatocyte growth factor detected in blood and bone marrow plasma of leukemia patients. *Br. J. Haematol.*, **87**, 640-642.

NAKAMURA S, GOHDA E, MATSUNAGA T, YAMAMOTO I AND MINOWADA J. (1994*b*). Production of hepatocyte growth factor by human haematopoietic cell lines. *Cytokine*, **6**, 285-294.

NAKAMURA T, TERAMOTO H AND ICHIHARA A. (1986). Purification and characterization of a growth factor from rat platelets for mature parenchymal hepatocytes in primary cultures. *Proc. Natl Acad. Sci. USA*, **83**, 6489-6493.

NAKAMURA T, NAWA K, ICHIHARA A, KAISE N AND NISHINO T. (1987). Purification and subunit structure of hepatocyte growth factor from rat platelets. *FEBS Lett.*, **224**, 311-316.

NAKAMURA T, NISHIZAWA T, HAGIYA M, SEKI T, SHIMONISHI M, SUGIMURA A, TASHIRO K AND SHIMIZU S. (1989). Molecular cloning and expression of human hepatocyte growth factor. *Nature*, **342**, 440-443.

NALDINI L, VIGNA E, NARSIMHAN RP, GAUDINO G, ZARNEGAR R, MICHALOPOULOS GK AND COMOGLIO PM. (1991*a*). Hepatocyte growth factor (HGF) stimulates the tyrosine kinase activity of the receptor encoded by the proto-oncogene c-MET. *Oncogene*, **6**, 501-504.

NALDINI L, WEIDNER KM, VIGNA E, GAUDINO G, BARDELLI A, PONZETTO C, NARSIMHAN RP, HARTMANN G, ZARNEGAR R, MICHALOPOULOS GK, BIRCHMEIER W AND CORMOGLIO PW. (1991*b*). Scatter factor and hepatocyte growth factor are indistinguishable ligands for the MET receptor. *EMBO J.*, **10**, 2867-2878.

NISHINO T, KAISE N, SINDO Y, NISHINO N, NISHIDA T, YASUDA S AND MASUI Y. (1991). Promyelocytic leukemia cell line, HL-60, produces human hepatocyte growth factor. *Biochem. Biophys. Res. Commun.*, **181**, 323-330.

NISHINO T, HISHA H, NISHINO N, ADACHI M AND IKEHARA S. (1995). Hepatocyte growth factor as a hematopoietic regulator. *Blood*, **85**, 3093-3100.

NOJI S, TASHIRO K, KOYAMA E, NOHNO T, OHYAMA K, TANIGUCHI S AND NAKAMURA T. (1990). Expression of hepatocyte growth factor gene in endothelial and Kupffer cells of damaged rat livers, as revealed by in situ hybridization. *Biochem. Biophys. Res. Commun.*, **173**, 42-47.

RUBIN JS, CHAN AM, BOTTARO DP, BURGESS WH, TAYLOR WG, CECH AC, HIRSCHFIELD DW, WONG J, MIKI T, FINCH PW AND AARONSON A. (1991). A broad-spectrum human lung fibroblast-derived mitogen is a variant of hepatocyte growth factor. *Proc. Natl Acad. Sci. USA*, **88**, 415-419.

RUSSELL WE, MCGOWAN JA AND BUCHER NL. (1984). Partial characterization of a hepatocyte growth factor from rat platelets. *J. Cell Physiol.*, **119**, 183-192.

SEKI T, IHARA I, SUGIMURA A, SHIMONISHI M, NISHIZAWA T, ASAMI O, HAGIYA M, NAKAMURA T AND SHIMIZU S. (1990). Isolation and expression of cDNA for different forms of hepatocyte growth factor from human leukocyte. *Biochem. Biophys. Res. Commun.*, **172**, 321-327.

SELDEN C, JONES M, WADE D AND HODGSON H. (1990). Hepatotropin mRNA expression in human foetal liver development and in liver regeneration. *FEBS Lett.*, **270**, 81-84.

TAJIMA H, MATSUMOTO K AND NAKAMURA T. (1991). Hepatocyte growth factor has potent anti-proliferative activity in various tumor cell lines. *FEBS Lett.*, **291**, 229-232.

TASHIRO K, HAGIYA M, NISHIZAWA T, SEKI T, SHIMONISHI M, SHIMIZU S AND NAKAMURA T. (1990). Deduced primary structure of rat hepatocyte growth factor and expression of the mRNA in rat tissues. *Proc. Natl Acad. Sci. USA*, **87**, 3200-3204.

WEIDNER KM, BEHRENS J, VANDEKERCKHOVE J AND BIRCHMEIER W. (1990). Scatter factor: molecular characteristics and effect on the invasiveness of epithelial cells. *J. Cell Biol.*, **111**, 2097-2108.

YANAGITA K, MATSUMOTO K, SEKIGUCHI K, ISHIBASHI H, NIHO Y AND NAKAMURA T. (1993). Hepatocyte growth factor may act as a pulmotrophic factor on lung regeneration after acute lung injury. *J. Biol. Chem.*, **268**, 21212-21217.

YANAGITA K, NAGAIKE M, ISHIBASHI H, NIHO Y, MATSUMOTO K AND NAKAMURA T. (1992). Lung may have an endocrine function producing hepatocyte growth factor in response to injury of distal organs. *Biochem. Biophys. Res. Commun.*, **182**, 802-809.

YUKIOKA K, INABA M, FURUMITSU Y, YUKIOKA M, NISHINO T, GOTO H, NISHIZAWA Y AND MORII H. (1994). Levels of hepatocyte growth factor in synovial fluid and serum of patients with rheumatoid arthritis and release of hepatocyte growth factor by rheumatoid synovial fluid cells. *J. Rheumatol.*, **21**, 2184-2189.